# Unlocking the antiviral potential of rosmarinic acid against chikungunya virus via IL-17 signaling pathway

**DOI:** 10.3389/fcimb.2024.1396279

**Published:** 2024-05-10

**Authors:** Xinfei Liao, Jialiang Xin, Ziping Yu, Weiming Yan, Chenghui Li, Liang Cao, He Zhang, Wei Wang

**Affiliations:** ^1^Wenzhou Polytechnic, Wenzhou, Zhejiang, China; ^2^Institute of Virology, Wenzhou University, Wenzhou, Zhejiang, China; ^3^College of Agriculture, Yanbian University, Yanji, Jilin, China; ^4^Changchun Veterinary Research Institute, Chinese Academy of Agricultural Sciences, Changchun, Jilin, China

**Keywords:** rosmarinic acid, chikungunya virus, IL-17 signaling pathway, network pharmacology, molecular docking

## Abstract

**Background:**

The Chikungunya virus is an *Alphavirus* that belongs to the *Togaviridae* family and is primarily transmitted by mosquitoes. It causes acute infection characterized by fever, headache, and arthralgia. Some patients also experience persistent chronic osteoarthritis-like symptoms. Dedicated antiviral treatments are currently unavailable for CHIKV. This study aims to explore the potential anti-CHIKV effect of rosmarinic acid using network pharmacology.

**Methods:**

This study employed network pharmacology to predict and verify the molecular targets and pathways associated with ROSA in the context of CHIKV. The analysis outcomes were further validated using molecular docking and *in vitro* experiments.

**Results:**

The analysis of CHIKV targets using the Kyoto Encyclopedia of Genes and Genomes and MCODE identified IL-17 as an important pathogenic pathway in CHIKV infection. Among the 30 targets of ROSA against CHIKV, nearly half were found to be involved in the IL-17 signaling pathway. This suggests that ROSA may help the host in resisting CHIKV invasion by modulating this pathway. Molecular docking validation results showed that ROSA can stably bind to 10 core targets out of the 30 identified targets. In an *in vitro* CHIKV infection model developed using 293T cells, treatment with 60 μM ROSA significantly improved the survival rate of infected cells, inhibited 50% CHIKV proliferation after CHIKV infection, and reduced the expression of TNF-α in the IL-17 signaling pathway.

**Conclusion:**

This study provides the first confirmation of the efficacy of ROSA in suppressing CHIKV infection through the IL-17 signaling pathway. The findings warrant further investigation to facilitate the development of ROSA as a potential treatment for CHIKV infection.

## Introduction

Chikungunya virus (CHIKV) infection, classified as an arboviral disease, was first reported in southern Tanzania in 1952 (Soumahoro et al., 2009). In recent years, it has emerged as a significant public health concern in tropical and subtropical regions (Rougeron et al., 2015). In China, the primary mode of the CHIKV epidemic is through importation from abroad. Recently, imported cases of CHIKV have been reported in several regions of South China, including Zhejiang Province, Guangdong Province, and Shenzhen City (Wu et al., 2012; Pan et al., 2019; Yang et al., 2020b; Su et al., 2023).

Interleukin-17 (IL-17) plays a vital role in CHIKV infection. Research indicates that CHIKV can elevate IL-17 levels during the acute phase of infection in patients (Mathew et al., 2017). Elevated IL-17 levels stimulate the production of factors such as IL-6, IL-8, and granulocyte colony-stimulating factor (G-CSF) (Gaffen, 2009). This pro-inflammatory environment triggers osteoclastogenesis, leading to bone loss and erosion (Mathew et al., 2017). Symptoms of the acute phase of CHIKV infection include fever, headache, nausea, myalgia, and arthralgia (Thiberville et al., 2013). While some individuals may recover without complications, many experience persistent chronic arthralgia, leading to a decline in social labor force participation (McCarthy et al., 2022). Specific antiviral treatments to combat CHIKV infection are currently unavailable (Chen and Wilson, 2012). Symptomatic management, primarily through nonsteroidal anti-inflammatory drugs (NSAIDs) and chloroquine, is commonly employed, albeit with potential side effects (Millsapps et al., 2022). Therefore, there is an urgent need for the development of effective anti-CHIKV drugs.

Rosmarinic acid (ROSA) is a phytochemical present in various plants, predominantly rosemary (*Rosmarinus officinalis*, Lamiaceae), as well as in other plants in the *Nepetoideae* subfamily of the *Lamiaceae*, such as sage, mint, thyme, lemon balm, basil, and oregano (Petersen and Simmonds, 2003). *In vitro* studies have demonstrated that ROSA exhibits multiple beneficial activities, including antioxidative, antibacterial, astringent, analgesic, anti-inflammatory, anticancer, cardioprotective, and neuroprotective properties (Amoah et al., 2016; Bekut et al., 2018; Elufioye and Habtemariam, 2019). Furthermore, ROSA has been found to have therapeutic effects on Osteoarthritis (OA). *In vitro* experiments have indicated that ROSA can inhibit degradation of the extracellular matrix (ECM) in OA by suppressing the production of Interleukin (IL)-6 and reversing the IL-1β-induced suppression of proteoglycan aggrecan (ACAN) and type 2 collagen (COL2) gene expression (Hu et al., 2018). Additionally, several studies have demonstrated that ROSA exhibits antiviral activity against various viruses, including influenza virus A (Jheng et al., 2022), Hepatitis B virus (Tsukamoto et al., 2018), and Dengue Virus (Samy et al., 2023).

**Figure 1:**
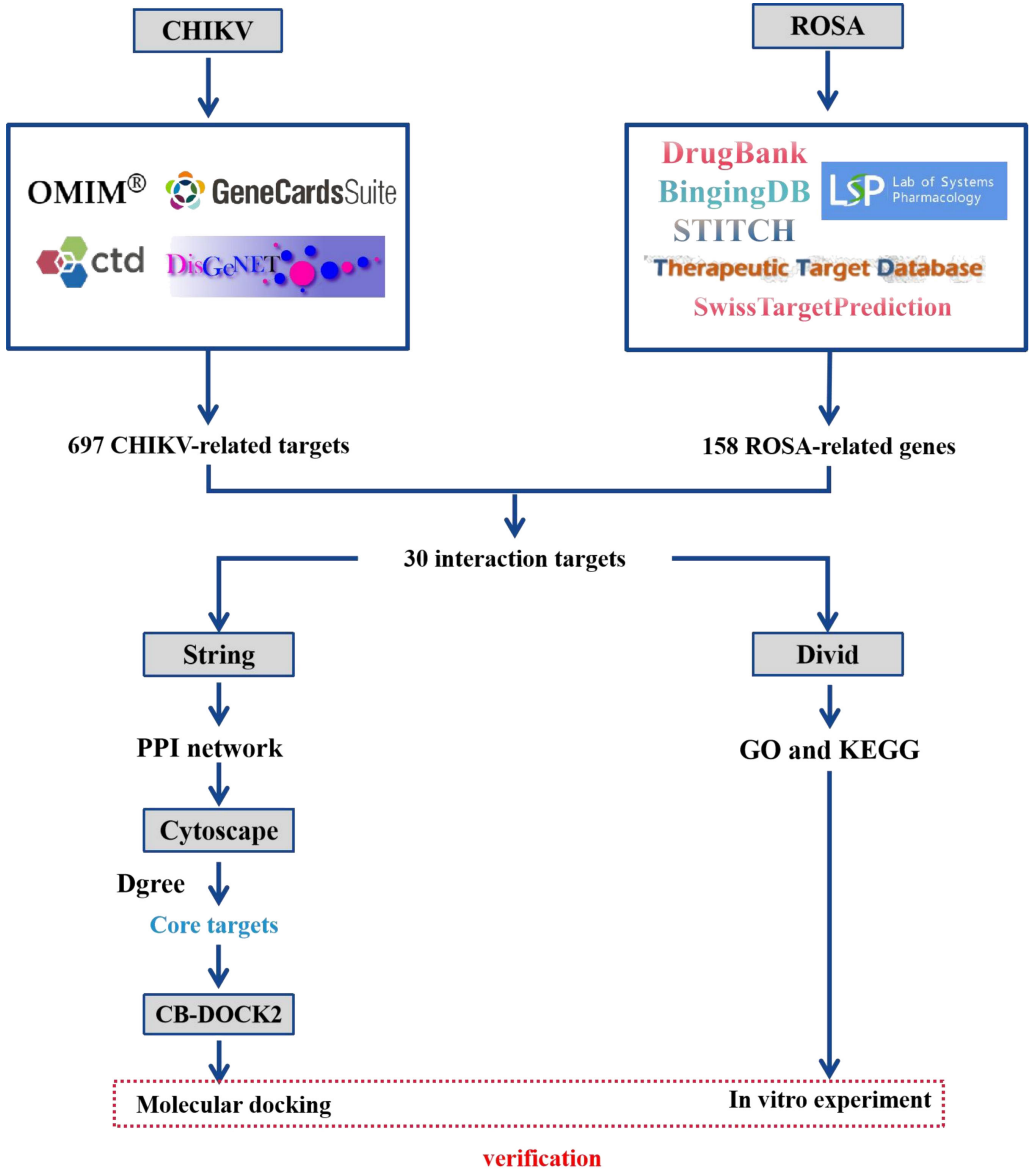
Flowchart of the study design.

Network pharmacology, introduced in 2007, is an emerging interdisciplinary field utilizing disease-gene-drug target networks to study the underlying biological mechanisms of drugs (Yang et al., 2013; Ru et al., 2014). By analyzing network interactions, network pharmacology provides valuable insights and scientific evidence for drug discovery, enabling a better understanding of the pharmacological mechanisms of active drug ingredients at the biomolecular level (Yang et al., 2013; Ru et al., 2014). From a network perspective, it offers a comprehensive and efficient approach to uncovering complex interactions between drugs and biological systems, including human organs, diseases, metabolic pathways, and target proteins (Davis et al., 2009; Zhang et al., 2021). This study aimed to characterize the potential molecular targets and signaling pathways involving ROSA in CHIKV infection treatment, while establishing a network to elucidate the interrelationships between ROSA and CHIKV targets. The workflow of this study is depicted in **Figure 1**.

## Materials and methods

### Cell lines, viruses and compounds

293T cells and BHK-21 cells were grown in Dulbecco’s modified Eagle’s medium (DMEM) (Gibco) containing 10% heat-inactivated fetal bovine serum (FBS) (Gibco), 100 units/mL of penicillin, 100 mg/mL of streptomycin in 5% CO2 at 37°C.

The CHIKV strain (GenBank: MT933041.1), was kindly provided by Changchun Veterinary Research Institute, was stored at -80°C until further use. Rosmarinic acid (ROSA) with a purity of ≥ 99.70% was obtained from MedChemExpress (CAS No.: 20283-92-5).

### Identification of disease-related targets

Disease-related targets were identified using “Chikungunya virus” and “Chikungunya fever” as keywords and retrieved from four databases: CTD (http://ctdbase.org), Disgenet (https://www.disgenet.org/), OMIM (https://omim.org/) and Genecards (https://www.genecards.org/), limited to the *Homo sapiens* organisms only. Then, the false-positive and duplicates were removed, and the data was integrated for further analysis.

### Prediction of the rosmarinic acid pharmacological target

The keyword “Rosmarinic acid” was used to retrieve relevant genes from six databases: TCMSPS (https://old.tcmsp-e.com/tcmsp.php), SwissTargetPrediction (http://www.swisstargetprediction.ch/), Drugbank (https://go.drugbank.com/), Binding database (http://www.bindingdb.org/bind/index.jsp), STITCH (http://stitch.embl.de/), and Therapeutic Target Database (http://db.idrblab.net/ttd/). The targets from these six databases were combined, and the repeat duplicate values were removed to obtain the comprehensive set of ROSA-related targets.

### Protein-protein interaction network analysis and the core targets screen

The overlapping targets of ROSA and CHIKV were obtained using jVenn (https://jvenn.toulouse.inrae.fr/app/example.html). Subsequently, the CHIKV target or potential targets of ROSA for CHIKV treatment was imported into the String database (https://string-db.org/), with the species “*Homo sapiens*” selected to obtain a protein-protein interaction (PPI) network. A confidence level of ≥0.9 was set for the interaction score. The data were then imported into the Cytoscape 3.9.1 software and Degree analysis was conducted to identify core proteins. Node size and color were adjusted based on the degree value. The final PPI network was visualized, and the MCODE plug-in of Cytoscape was utilized to analyze the key subnetworks, focusing on the important topological sub-network structures. The top three significant subnetworks were further analyzed by using the Kyoto Encyclopedia of Genes and Genomes (KEGG) analysis.

### Gene Ontology and Kyoto Encyclopedia of genes and genomes enrichment analyses

To elucidate the involvement of potential ROSA targets in CHIKV treatment, the potential targets were subjected to DAVID (https://david.ncifcrf.gov/tools.jsp) for Gene Ontology (GO) and Kyoto Encyclopedia of Genes and Genomes (KEGG) analyses. The obtained data were saved, and the top 20 significantly enriched results were selected for further visual analysis.

### Molecular docking verification

To validate the binding affinity of ROSA with CHIKV target proteins and assess its therapeutic potential, molecular docking analyses were conducted on the core target proteins. The three-dimensional (3D) structures of the target proteins were retrieved from the Protein Data Bank (PDB) database (https://www.rcsb.org), specifically limited to *Homo sapiens*. Protein conformations in 3D protein with a crystal resolution of < 3, determined using X-ray crystal diffraction, were obtained by searching the PDB (https://www.rcsb.org) for the target genes identified in the first ten PPI findings.

The two-dimensional (2D) structure of ROSA was obtained from the PubChem website. Subsequently, the protein and ligand structures were imported into the CB-DOCK2 (http://cadd.labshare.cn/cbdock2/php/index.php) for Structure-based Blind Docking analysis, with visualization of the docking results based on the highest Vina score.

### Evaluation of the protective effect of ROSA in 293T cells infected with CHIKV

To determine the Median Cytotoxic Concentration (CC50) of ROSA on 293T cells, cells were seeded in a 96-well plate and exposed to eight different concentrations of ROSA (3.75 µM, 7.5 µM, 15 µM, 30 µM, 60 µM, 125 µM, 250 µM, and 500 µM) for 48 h. Cell viability was assessed using CCK-8 assay.

To study the protective effect of ROSA on 293T cells infected with CHIKV, cells were cultured in 96-well plates and infected with CHIKV at a MOI of 0.01 for 1 h (Ho et al., 2018). After infection, the culture medium was replaced with 60 µM ROSA, and cells were further incubated for 48 h. The CCK-8 assay was performed to evaluate the inhibitory impact of ROSA on virus-induced cellular damage.

To explore the impact of ROSA on CHIKV replication, 293T cells were seeded in 48-well plates and infected with a mixture of 60 µM ROSA and 1 MOI CHIKV for 1 h (Lani et al., 2015). The inoculum was then replaced with 60 μM ROSA and incubated for another 48 h. The supernatant was collected for RT-qPCR detection. Furthermore, for a better presentation of the ROSA inhibition of viral plaques, collected supernatant was re-infected into BHK-21 cells, followed by crystal violet staining. The nsp2 primers were used (Ho et al., 2010) and the primer sequences were in **Supplementary Table 1**.

To determine the steps in the viral life cycle that are inhibited by ROSA, time-addition experiments were performed. 293T cells in 48-well plates were infected with CHIKV at an MOI of 0.01 for 1 h (Ho et al., 2018). The inoculum was then replaced with fresh culture media and incubated for an additional 48 h. ROSA (60 μM) was added during the 1h pre-absorption period (pre-infection), absorption period (co-treatment), or post-absorption period (post-infection). Supernatants were collected and RNA was extracted for RT-qPCR assays.

### Antiviral replication assay and targets validation of ROSA *in vitro*

To investigate the impact of ROSA on the IL-17 signaling pathway in the context of CHIKV infection, a 293T cell monolayer was cultured in a 48-well plate. The 60 μM ROSA were added to the wells along with CHIKV at an MOI of 1 for a 48-h incubation period (Lani et al., 2015). Subsequently, supernatants were collected, and RNA was extracted for RT-qPCR assays of TNF-α, CASP3, and MAPK8. Primer sequences were designed based on previous reports (Peng et al., 2019; Yang et al., 2020a; Yang et al., 2022), as depicted in **Supplementary Table 1**. Additionally, the RNA abundance was measured with the 2^−ΔΔCt^ method.

### Statistical analysis

The experimental data underwent statistical analysis using GraphPad Prism 5.0. Significance levels were established using either the unpaired Student’s t-test for two-group comparisons or one-way analysis of variance (ANOVA) for multiple group comparisons (*P* < 0.05 indicated statistical significance).

## Results

### Acquisition of the targets for ROSA and CHIKV

A total of 292 genes and 137 genes were identified from CTD and Disgenet databases, respectively, based on the criterion of inference score > 0. Additionally, a total of 405 targets were obtained from the GeneCards database. A total of 192 related genes were identified following a search in the OMIM database. Combining these results and eliminating duplicate genes, a comprehensive list of 697 CHIKV-related targets was compiled.

Furthermore, a search of various databases including TCMSPS, SwissTargetPrediction, Drugbank, Binding database, STITCH, and Therapeutic Target Database was performed. Using the term “Rosmarinic acid”, 158 genes associated with ROSA were collected after eliminating duplicate targets.

### Acquisition and analysis of intersection targets of ROSA and CHIKV-

**Figure 2:**
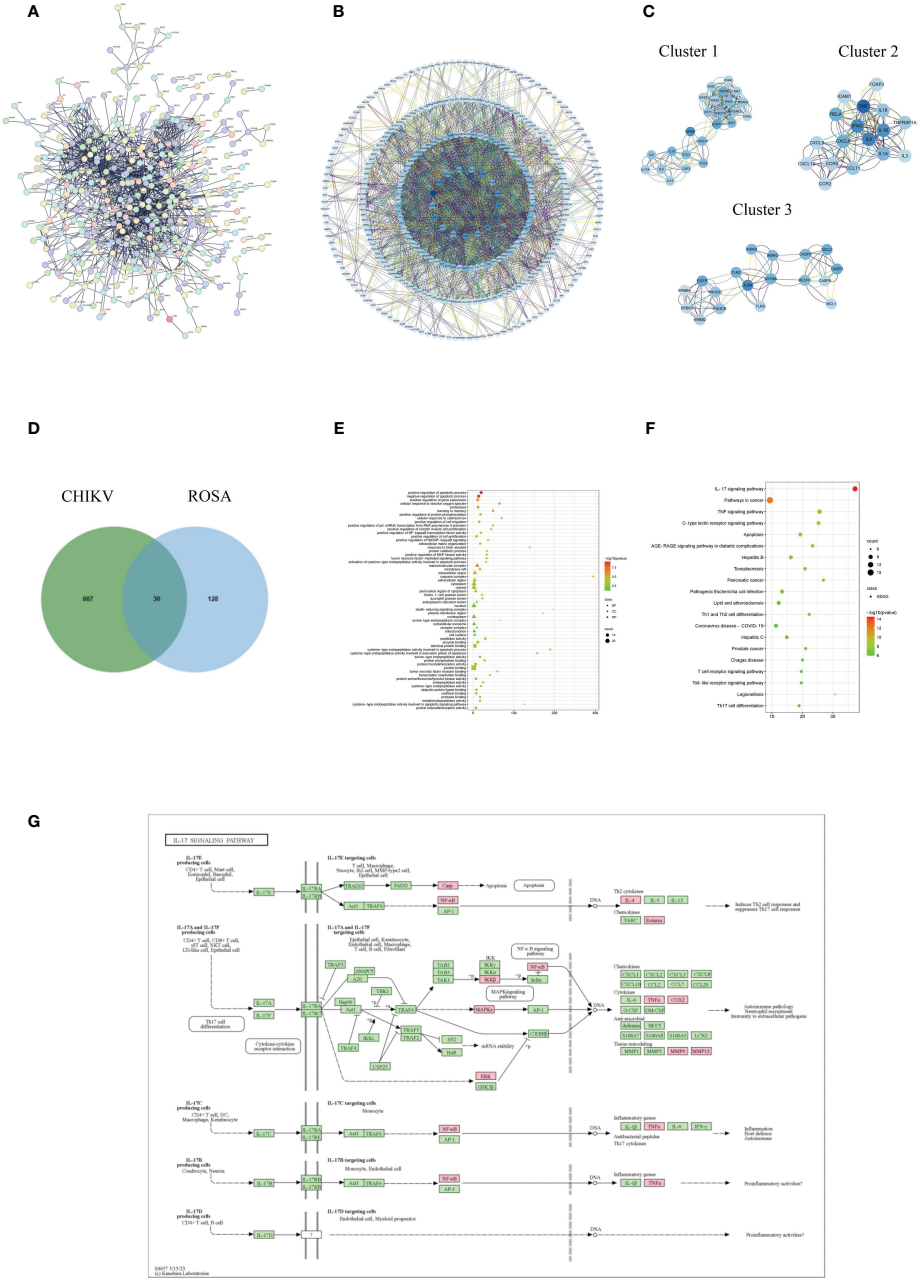
Acquisition and analysis of intersection targets of ROSA and CHIKV **(A)** PPI network of the CHIKV targets. **(B)** Analysis of core targets of CHIKV by degree analysis using Cytoscape software. The more important the gene, the darker its marking color. **(C)** Top three functional clusters in CHIKV by MCODE analysis using Cytoscape. **(D)** Intersection of ROSA targets and CHIKV targets resulting in 30 common genes. **(E)** Bubble chart of GO biological function analysis of 30 intersection genes. **(F)** Bubble chart of KEGG pathway analysis of 30 intersection genes. **(G)** Diagram of ROSA’s resistance to CHIKV in the IL-17 signaling pathway. Pink represents being influenced by ROSA.

The PPI analysis of the CHIKV disease target network revealed a total of 502 nodes and 1,386 edges, with a highly significant p-value of < 1.0e-16 (**Figure 2A**). Core targets such as TP53, TNF, IL-6, IFNG, TLR4, AKL1, IL-1B, and *NFKB1* were identified based on their degree scores (**Figure 2B**), forming three major functional clusters (**Figure 2C**). KEGG analysis results showed differences in the functions of these three clusters, but all showed enrichment in the IL-17 signaling pathway (**Table 1**). The intersection of ROSA targets and CHIKV targets resulted in 30 common targets (**Figure 2D**), showcasing a strong relationship with the disease network, including key CHIKV targets like TNF, STAT1, and CASP3.

**Table 1:** MCODE enrichment analysis of CHIKV-related protein targets.

Cluster	Count	Genes	GO	Description	PValue
1	24	IL17A, CXCL10, IFI44L, ISG15, DDX58, STAT1, MX1, IL13, IFIT1, RSAD2, OAS1, CSF2, IFIH1, IRF7, NFKB1, IL4, EIF2AK2, OAS2, CCL2, OASL, IL2, OAS3, IL10, CCL5	hsa04657	IL-17 signaling pathway	6.97E-08
			hsa04621	NOD-like receptor signaling pathway	1.08E-07
			hsa04060	Cytokine-cytokine receptor interaction	1.37E-06
2	17	ICAM1, CCR2, IL18, CXCL9, IL3, CXCL12, FOXP3, CXCL8, IL1B, IL1A, TNFRSF1A, IFNG, TNF, RELA, CCL11, IL6, CCR5	hsa04060	Cytokine-cytokine receptor interaction	3.97E-17
			hsa04061	Viral protein interaction with cytokine and cytokine receptor	2.92E-14
			hsa04657	IL-17 signaling pathway	1.07E-08
3	18	TLR4, MYD88, PIK3CB, BECN1, IKBKG, EGFR, ERBB2, IKBKB, TLR2, CASP9, CASP3, CASP8, TLR9, ERBB3, MCL1, ERBB4, BCL2, PIK3CD	hsa04151	PI3K-Akt signaling pathway	1.22E-13
			hsa04620	Toll-like receptor signaling pathway	1.07E-11
			hsa04657	IL-17 signaling pathway	7.73E-04

GO enrichment analysis provided insights into functions in three categories: biological process (BP), cellular component (CC), and molecular function (MF). The analysis of the 30 intersection targets yielded a total of 232 items. The top 20 significantly enriched terms in BP, MF, and CC categories were selected based on a significance level of *P* < 0.05 (**Figure 2E**).

In the BP category, target proteins were prominently associated with processes such as positive regulation of the apoptotic process, negative regulation of the apoptotic process, and positive regulation of gene expression. In the MF category, the target proteins were mainly involved in peptidase activity, enzyme binding, and identical protein binding. In the CC category, the target proteins were classified into the macromolecular complex, membrane raft, and extracellular space.

A total of 108 KEGG enrichment items were identified, and the top 20 items were screened based on the KEGG analysis with a significance level of *P* < 0.05. The IL-17 signaling pathway emerged as the most enriched pathway (**Figure 2F**). Among the genes associated with the highest number of pathways, notable candidates included MAPK8, CASP8, MAPK1, PTGS2, MMP9, and RELA (**Table 2**). This suggests that the IL-17 signaling pathway may be the most important pathway through which ROSA exerts its therapeutic effects on CHIKV (**Figure 2G**).

**Table 2:** Enrichment pathways corresponding to the intersection genes.

Category	Term	Dsecription	Count	Genes	PValue
KEGG	hsa04657	IL-17 signaling pathway	12	IL4, IKBKB, MAPK8, MMP13, CCL11, CASP8, CASP3, MAPK1, PTGS2, TNF, MMP9, RELA	3.15E-15
KEGG	hsa05200	Pathways in cancer	17	STAT1, MMP2, F2, PTGS2, MMP9, EGFR, IL2, RELA, IL4, IKBKB, AR, CASP7, MAPK8, CASP8, CASP3, ERBB2, MAPK1	6.95E-13
KEGG	hsa04668	TNF signaling pathway	10	IKBKB, CASP7, MAPK8, CASP8, CASP3, MAPK1, PTGS2, TNF, MMP9, RELA	5.90E-11

### Core target analysis of 30 intersection genes and molecular docking

**Figure 3:**
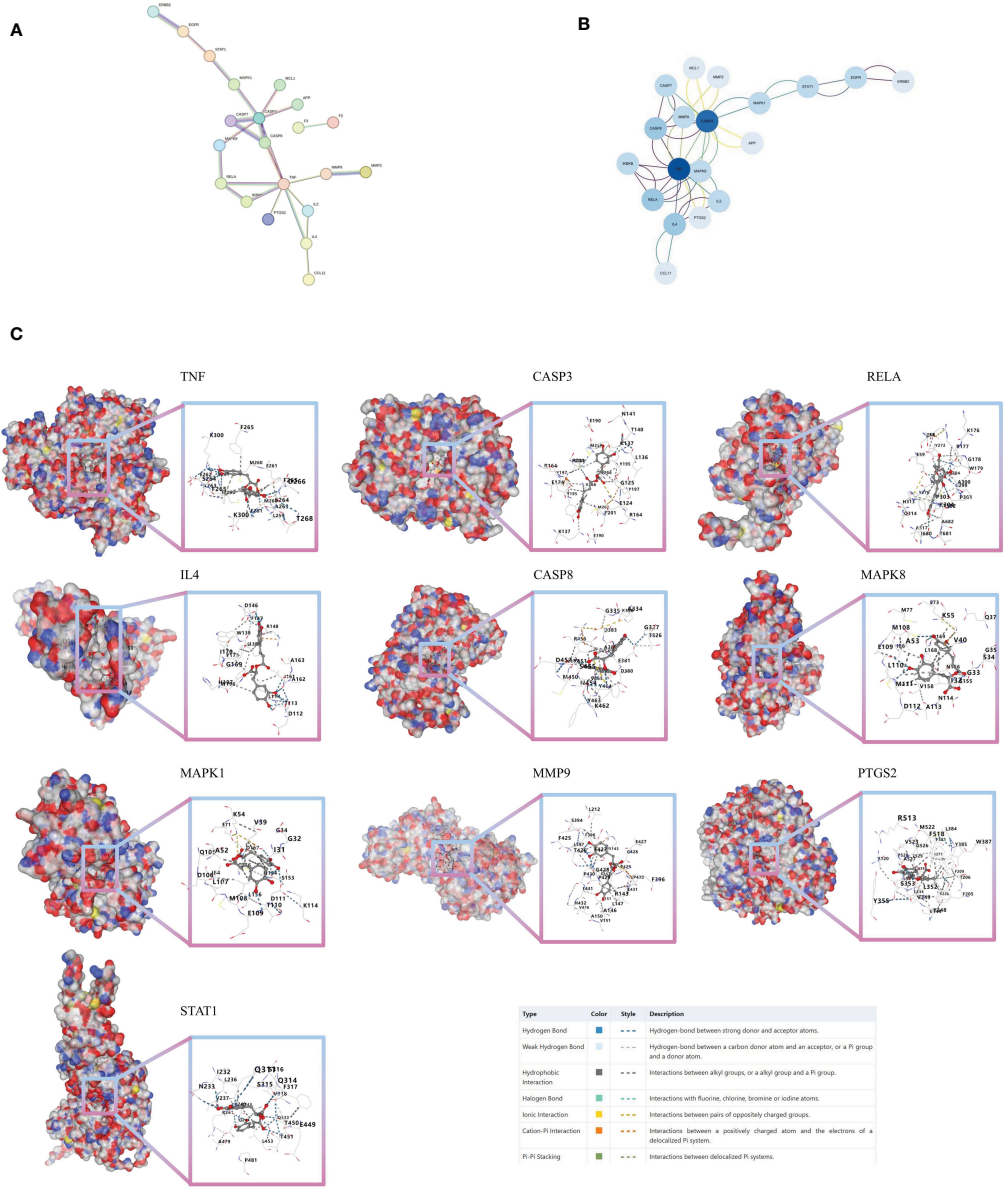
Molecular docking verification **(A)** PPI network of 30 intersection genes of ROSA and CHIKV. **(B)** Analysis of core targets of 30 intersection genes of ROSA and CHIKV. **(C)** Molecular models of ROSA binding to its predicted protein targets.

From the 30 associated targets between ROSA and CHIKV, 21 targets were selected using STRING (**Figure 3A**). Significant high-degree nodes in the interaction network were identified through degree analysis in Cytoscape 3.9.1.software, including TNF, CASP3, RELA, IL4, and CASP8 (**Figure 3B**).

Next, we tested the following potential target proteins: TNF (5e1t), CASP3 (7rn7), RELA (6hl6), IL4 (1iar), CASP8 (3h11), MAPK8 (2xrw), MAPK1 (6g54), MMP9 (5th6), PTGS2 (5d19), and STAT1 (8d3f). The results showed that ROSA could stably bind to 10 target proteins, with binding scores consistently below -8 (**Figure 3C**; **Supplementary Table 2**).

### ROSA enhances the viability of 293T cells infected with CHIIKV

**Figure 4:**
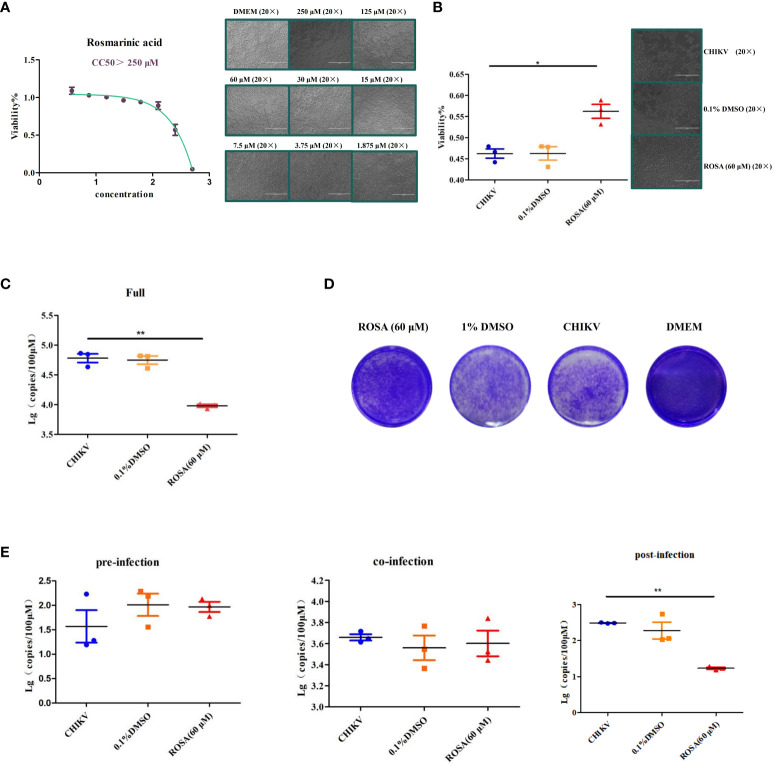
Enhancement of the viability of 293T cells infected with CHIIKV by ROSA. **(A)** CC50 of ROSA. Microscopic observation of 293T cells when treated with ROSA at eight different concentrations. **(B)** ROSA inhibits 293T cell damage induced by CHIKV infection. Microscopic observation of 293T cells infected with CHIKV when treated with ROSA (20 × magnification). **(C)** Inhibitory effect of ROSA on CHIKV. **(D)** Crystal violet staining results in BHK-21 cells. **(E)** Intervention effect of ROSA on the virus lifecycle in 293T cells. All the data are compared with the CHIKV group. All values are presented as mean ± SD. **P < 0.01, *P < 0.05.

In order to determine the optimal safe dosage of ROSA for 293T cells, the cytotoxicity of ROSA was assessed at concentrations ranging from 1 to 500 μM using the CCK-8 assay. This assessment, conducted independently three times at 48 h post-treatment, revealed that the calculated 50% cytotoxic concentrations exceeded 250 μM at 48 h, with 60 μM identified as the maximum non-toxic dose for 293T cells (**Figure 4A**).

Subsequently, the protective effect of ROSA on 293T cells infected with CHIKV, was investigated using the CCK-8 assay over a 48 h period. The results showed that 60 μM ROSA significantly inhibited the damage of 293T cells infected with 0.01 MOI CHIKV within 48 h. Notably, the solvent of 1% DMSO failed to impede the damage of 293T cells (**Figure 4B**).

The results of RT-qPCR showed that 60 μM ROSA significantly reduced the replication of CHIKV in 293T cells (**Figure 4C**), whereas 0.1% DMSO had no antiviral effect. Moreover, crystal violet staining results showed that 60 μM ROSA could significantly decrease the number of plaques of CHIKV in BHK-21 (**Figure 4D**).

To further determine the impact of ROSA on the viral life cycle, a time-of-addition assay was performed. The results showed that 60 μM ROSA has no effect on CHIKV replication during the pre- and co-infection of 293T cells. However, post-infection, ROSA exhibited a significant inhibitory effect on the proliferation of CHIKV in 293T cells, resulting in an approximately 50% decrease in the viral copy number at 48 h (**Figure 4E**).

### Validation of targets by RT-qPCR

**Figure 5:**
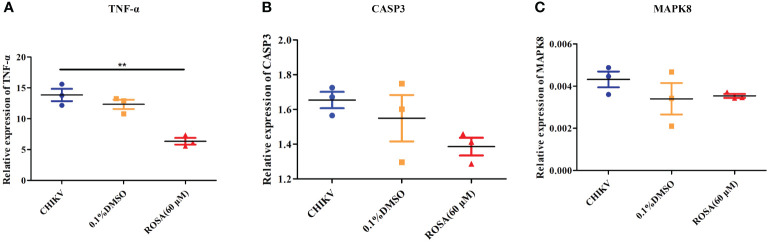
Validation of targets by qRT-PCR **(A)** Relative expression of TNF-α. **(B)** Relative expression of CASP3. **(C)** Relative expression of MAPK8. All the data are compared to the cell group infected with CHIKV. All values are presented as mean ± SD. **P < 0.01.

An investigation into the expression of TNF-α, CASP3, and MAPK8 in 293T cells under different incubation conditions revealed that 0.1% DMSO could not influence the expression of TNF-α, CASP3, and MAPK8 in CHIKV-infected 293T cells. However, treatment with 60 μM ROSA significantly reduced the expression of TNF-α in 293T cells infected with CHIKV at an MOI of 1 (**Figures 5A–C**).

## Discussion

The global dissemination of CHIKV has been shaped by genetic variations within the virus (Vega-Rúa et al., 2020). Initially limited to tropical and subtropical regions due to the prevalence of *Aedes aegypti* mosquitoes, the virus expanded its range following a mutation of CHIKV (E1-A226V) that enabled transmission by *Aedes albopictus* mosquitoes. This genetic adaptation allowed CHIKV to thrive in temperate regions, leading to its broader geographical spread (Constant et al., 2021; Bettis et al., 2022).

Effective treatment for CHIKV is currently lacking, with supportive care being the main approach (Staples et al., 2009). While over-the-counter NSAIDs are commonly used to relieve joint pain and reduce fever, they may not be suitable for individuals with hematological diseases (Amaral et al., 2020b). Chloroquine, used in some countries, has shown no efficacy in addressing viremia and viral load (Javelle et al., 2015; Burt et al., 2017). Therefore, the imperative to develop safe and effective therapeutic drugs for CHIKV remains a crucial aspect of public health.

Network pharmacology serves as a valuable tool in drug discovery, providing insights into the interaction between drugs and diseases. In this study, bioinformatics methods were employed to identify key targets associated with CHIKV infection. Notable targets such as TP53, TNF, IL-6, IFNG, TLR4, AKL1, IL-1B, and NFKB1 were identified and organized into three main clusters that regulate the expression of other genes. KEGG analysis revealed the involvement of the IL-17 signaling pathway in all three clusters, highlighting its significance as a mechanism for CHIKV-related diseases in the host.

A previous studies have shown increased levels of IL-17 in patients infected with CHIKV (Javelle et al., 2015). Elevated levels of IL-17 are associated with various autoimmune diseases, including rheumatoid arthritis (Kuwabara et al., 2017). Chronic arthritis caused by CHIKV shares symptomatic similarities with rheumatoid arthritis, classifying it as an autoimmune disease (Amaral et al., 2020a). IL-17 may contribute to matrix and bone destruction by stimulating IL-6, TNF, IL-1B, matrix metalloproteinases, and the nuclear activator receptor kB-nuclear activator receptor kB ligand (RANKL) system (Miossec et al., 2009). Elevated IL-6 levels further activate RANKL and inhibit osteoprotegerin release, exacerbating bone damage (Noret et al., 2012). Additionally, IL-17A impedes the expression of IFN-α2, prolonging the presence of CHIKV in certain patients (Neupane et al., 2020). This sustained viral presence enables continuous damage of the host’s bones through the IL-17 signaling pathway. Based on these findings, targeting the IL-17 signaling pathway emerges as a promising therapeutic approach for CHIKV-related diseases. Further exploration of interventions regulating this pathway may hold the potential to prevent or alleviate the detrimental effects of CHIKV infection.

In the present study, an in-depth analysis of the intersection between ROSA targets and CHIKV targets was conducted, identifying 30 targets that have a strong relationship within the disease network. These targets include key CHIKV targets such as TNF, STAT1, and CASP3. KEGG enrichment analysis revealed the IL-17 signaling pathway as a central mechanism via which ROSA exerts its effects on CHIKV. The IL-17 signaling pathway map demonstrated that ROSA acts on multiple targets and establishes interactive relationships with apoptosis, the NF-κB signaling pathway, and the MAPK signaling pathway.

Furthermore, GO enrichment analysis of the 30 identified genes revealed that ROSA influences biological processes through macromolecular complexes, membrane rafts, and extracellular space. This impact extends to peptidase activity, enzyme binding, and identical protein binding, ultimately contributing to the positive regulation of apoptotic processes, negative regulation of apoptotic processes, and positive regulation of gene expression. To validate the reliability of the ROSA-CHIKV target intersection, molecular docking was employed, revealing consistently low Vina scores below -8, indicating stable binding of ROSA to these proteins. This interaction may potentially counteract the damage caused by CHIKV infection.

To further confirm the impact of ROSA on the IL-17 signaling pathway in CHIKV treatment, a comparison was made between the expression levels of TNF-α, CAPS3, and MAPK8 in the IL-17 signaling pathway of 293T cells infected with CHIKV versus 293T cells co-incubated with ROSA and CHIKV. The results showed that 60 μM ROSA significantly reduced the expression of TNF-α in the IL-17 signaling pathway. TNF-α plays a critical role in immune-inflammatory diseases like rheumatoid arthritis, and symptom relief in patients can be effectively achieved by inhibiting TNF-α (Prado et al., 2017). While the exact mechanism of CHIKV-induced arthritis remains unclear, emerging evidence suggests similarities between its mechanism and that of rheumatoid arthritis (Kumar et al., 2021). Numerous studies have shown that the levels of TNF-α in the serum of CHIKV-infected hosts significantly increase (Phuklia et al., 2013; Nayak et al., 2017). In this study, it was observed that ROSA could lower TNF-α expression levels, suggesting that ROSA may have the potential to alleviate CHIKV-induced arthritis. However, further tests are needed. A previous study has reported that the IL-17 signaling pathway promotes the replication of CHIKV (Dhanwani et al., 2012), suggesting that ROSA not only influences the resilience of gene targets against CHIKV-induced damage but may also exhibit an inhibitory effect on the replication of CHIKV. To validate this hypothesis, we first added 60 μM ROSA to 293T cells, allowing ROSA to engage in various stages of CHIKV replications. The collected supernatants were subsequently reinfected with BHK-21 cells, followed by crystal violet staining. Experimental findings confirmed that ROSA could significantly inhibit the replication of CHIKV within the cells. The results of the time-of-addition assay further demonstrated ROSA had a significant inhibitory effect on the proliferation of CHIKV in 293T cells, resulting in a nearly 50% decrease in the viral copy number at 48 h post-infection. However, in both the pre-treatment group and the co-infection groups, no difference in the viral copy number between the ROSA-treated and the CHIKV-only group. Based on the above results, it can be inferred that the inhibitory effect of ROSA on CHIKV replication may occur in the later stages of CHIKV replication, but the compound lacks influence on the early viral adsorption stage.

In conclusion, our study employed a combination of network pharmacology and cellular experiments, demonstrating the promising potential of ROSA in CHIKV treatment. However, our study has certain limitations. Our investigation focused solely on the therapeutic effects of ROSA against CHIKV in 293T cells, with the antiviral efficacy *in vivo* remaining unverified. Future studies will supplement *in vivo* antiviral drug experiment to enhance our comprehensive understanding of the therapeutic effects of ROSA against CHIKV.

## Data availability statement

The original contributions presented in the study are included in the article/**Supplementary Material**. Further inquiries can be directed to the corresponding authors.

## Ethics statement

Ethical approval was not required for the studies on humans in accordance with the local legislation and institutional requirements because only commercially available established cell lines were used. Ethical approval was not required for the studies on animals in accordance with the local legislation and institutional requirements because only commercially available established cell lines were used.

## Author contributions

XL: Data curation, Supervision, Writing – original draft, Software. JX: Validation, Writing – original draft. ZY: Validation, Writing – original draft, Supervision. WY: Validation, Writing – original draft. CL: Validation, Writing – original draft, Methodology. LC: Methodology, Writing – original draft, Funding acquisition. HZ: Methodology, Writing – review & editing, Supervision, Writing – original draft. WW: Funding acquisition, Methodology, Project administration, Supervision, Writing – review & editing, Writing – original draft.
