# Supplementary material for: Unlocking the antiviral potential of rosmarinic acid against chikungunya virus via IL-17 signaling pathway
Source: Front Cell Infect Microbiol. 2024 May 10;14:1396279. doi: 10.3389/fcimb.2024.1396279 (PMC11127627; doi:10.3389/fcimb.2024.1396279)
Supplement: Supplementary file 1 [file Table_1.docx]

**Supplementary table 1**

Table. 1 primer pairs for Identification

| Primers | Sequence |
| --- | --- |
| nsp2 | 5′-GGCAGTGGTCCCAGATAATTCAAG-3′ |
|  | 5′-GTACATACCCCACCTAGATCTGTCG-3′ |
| CASP3 | F：5′-AGCCCATTTCTCCATACG-3′ |
|  | R：5′-TTATTGCCTCACCACCTTTAG-3′ |
| TNFα | F：5′-CCTCTCTC TAATCAGCCCTCTG-3′ |
|  | R：5′-GAGGACCTGGGAGTAGTAGTAGGAG-3′ |
| MAPK8 | F：5′-AGGACTGCAGGAACGAGTTTT-3′ |
|  | R：5′-TAGCCCATGCCAAGGATGAC-3′ |
| GAPDH | F：5′-GCACCGTCAAGGCTGAGAAC-3′ |
|  | R：5′-TGGTGAAGACGCCAGTGGA-3′ |
