# Supplementary material for: Unlocking the antiviral potential of rosmarinic acid against chikungunya virus via IL-17 signaling pathway
Source: Front Cell Infect Microbiol. 2024 May 10;14:1396279. doi: 10.3389/fcimb.2024.1396279 (PMC11127627; doi:10.3389/fcimb.2024.1396279)
Supplement: Supplementary file 2 [file Table_2.docx]

Supplementary Table 2. Molecular docking-related information

| Target | Vina score | Contact residues | | | | |
| --- | --- | --- | --- | --- | --- | --- |
|  |  | Chain A | Chain B | Chain C | Chain D | Chain S |
| TNF | -8.7 | MET260 GLU261 PHE265 LYS300 | MET260 GLU261 GLU262 ALA263 SER264 PHE265 LYS300 | LEU259 MET260 GLU261 ALA263 SER264 PHE265 ASP266 THR268 |  |  |
| CASP3 | -8.2 | GLU124 LYS137 ARG164 | GLU190 TYR195 TYR197 PRO201 CYS264 VAL266 MET268 | GLU124 GLY125 LEU136 LYS137 THR140 ASN141 ARG164 | GLU190 TYR195 TYR197 PRO201 VAL266 MET268 |  |
| RELA | -7.2 | ILE56 GLU57 GLU59 LYS176 ARG177 GLY178 TRP179 GLN204 TYR272 MET275 TYR276 GLY299 ALA300 PRO301 THR302 PRO303 LYS304 HIS313 GLN314 ALA317 |  |  |  | ILE680 THR681 ALA682 |
| IL-4 | -7.7 | TYR56 ARG81 LYS84 ARG88 | TYR37 GLN38 LEU39 PHE41 LEU42 LEU43 SER44 GLU45 ALA46 HIS47 MET65 ASP66 ASP67 TYR74 |  |  |  |
| CASP8 | -8.8 | ASN447 MET450 TYR451 ASP452 ASN454 SER455 LYS462 TYR463 TYR464 VAL465 TRP466 | THR326 GLY327 ALA334 GLY335 LYS338 ASP380 GLU381 ALA382 ASP383 THR454 ARG456 |  |  |  |
| MAPK8 | -8.4 | ILE32 GLY33 SER34 GLY35 GLN37 VAL40 ALA53 LYS55 GLU73 MET77 ILE86 MET108 GLU109 LEU110 MET111 ASP112 ALA113 ASN114 SER155 ASN156 VAL158 LEU168 ASP169 |  |  |  |  |
| MAPK1 | -8 | ILE31 GLY32 GLY34 VAL39 ALA52 LYS54 GLU71 ILE84 GLN105 ASP106 LEU107 MET108 GLU109 THR110 ASP111 LYS114 SER153 ASN154 LEU156 CYS166 ASP167 |  |  |  |  |
| MMP9 | -8.4 | ARG143 LEU147 VAL151 LEU212 SER394 PHE396 LEU397 PHE425 THR426 GLU427 GLY428 PRO429 PRO430 LEU431 HIS432 VAL436 | ARG143 ALA146 LEU147 ALA150 VAL151 PHE396 GLU427 GLY428 PRO429 PRO430 LEU431 |  |  |  |
| PEGS2 | -9.7 | ARG120 PHE205 THR206 PHE209 VAL228 VAL344 TYR348 VAL349 LEU352 SER353 TYR355 ASN375 ILE377 PHE381 LEU384 TYR385 TRP387 ARG513 PHE518 MET522 VAL523 GLY526 ALA527 PRO528 PHE529 LEU531 LYS532 GLY533 LEU534 |  |  |  |  |
| STAT1 | -7.5 | ILE232 ASN233 LEU236 VAL237 LYS240 ARG241 GLN243 GLN244 GLN311 GLN314 SER315 SER316 PHE317 VAL318 GLN322 GLU449 THR450 THR451 LEU453 ALA479 PRO481 |  |  |  |  |
